# Acceptability and feasibility of strategies to promote healthy dietary choices in UK secondary school canteens: a qualitative study

**DOI:** 10.1186/s13104-021-05778-3

**Published:** 2021-09-20

**Authors:** Marie Murphy, Daniel Mensah, Elena Mylona, Oyinlola Oyebode

**Affiliations:** 1 Warwick Medical School, University of Warwick, Coventry, CV4 7AL UK; 2 Present Address: Murray Learning Centre, Institute of Applied Health Research, University of Birmingham, Birmingham, B15 2TT UK

**Keywords:** Choice architecture, Diet, Adolescents, Schools, Qualitative

## Abstract

**Objective:**

To explore the acceptability and feasibility of choice architecture strategies for dietary change in UK secondary school canteens from the perspectives of pupils, school staff and catering providers through qualitative focus groups and interviews.

**Results:**

Three focus groups with adolescents (n = 15; mean age 13.7 years; standard deviation 1.9) and eight interviews with school staff and caterers recruited from one school and catering provider in Coventry UK were undertaken. The most acceptable choice architecture strategies for intervening to drive healthy dietary choices are those that make use of proximity and positioning, on the basis that convenience was one of the main drivers for food/drink selections. Acknowledging adolescents’ desire for autonomy and for food to be familiar and predictable was considered important in enhancing acceptability. Challenges to the feasibility of nudge strategies included concerns about behavioural issues, increased food waste, and a decline in uptake of canteen purchases. The design of food choice architecture interventions for secondary school settings should consider the specific characteristics of this age group and setting to ensure successful implementation.

**Supplementary Information:**

The online version contains supplementary material available at 10.1186/s13104-021-05778-3.

## Introduction

This research aimed to qualitatively explore the acceptability and feasibility of food choice architecture in a secondary school canteen, from the perspectives of pupils, staff and caterers. The objectives were to investigate:

1. Perspectives on choice architecture
2. Influences upon pupils’ food choices in their school canteen
3. Opportunities and challenges facing schools in creating a healthy school canteen
4. Attitudes towards specific nudge strategies and healthy eating messages

### Background

Choice architecture (also known as ‘nudge’) is a behaviour change approach in which proximal physical micro-environments are altered to cue healthier behaviour [1]*.* Choice architecture may prove an effective means of changing dietary behaviours in adolescents in secondary schools given its effectiveness in other school and university settings [2]. However there is a lack of literature on attitudes towards nudge strategies despite the importance of attitudes in planning and evaluating interventions, including their acceptability, feasibility, economic viability, and theoretical underpinnings [3].

## Main text

### Materials and methods

This qualitative research consisted of focus groups (FGs) with pupils aged 11–18 years; and interviews with school staff, recruited from one secondary school in Coventry, UK. A school teacher invited potential participants and distributed information sheets (including aims of the study and reason for doing the research). The teacher was asked to invite adolescents representative of school demographics (age, sex, ethnicity, socioeconomic status) and staff participants in senior leadership, catering and pastoral roles. All those invited agreed to participate, however one adolescent did not have parental consent so was unable to take part.

Participants completed a questionnaire to collect demographic data (e.g. postcode, ethnicity, gender, age, job role). FGs and interviews were held on the school site, except for three telephone interviews (with staff). The facilitator (MM) used a semi-structured topic guide (developed by the authors; Additional file 1), and a second researcher (DM) took notes (during face-to-face data collection only). MM is a female Research Fellow with formal training and several years’ experience in qualitative research methods. Card-sorting activities were used to understand attitudes towards specific nudge strategies (see Table 3) and healthy eating messages (Additional file 2). The research was guided by constructivist and pragmatic orientations.

#### Data analysis

FGs and interviews were audio recorded, transcribed then anonymised. All data were analysed using thematic framework analysis [4] in NVivo v12. Exploratory inductive double-coding of a sample of transcripts was undertaken (MM, OO and DM) followed by a meeting to agree a coding framework, to enhance the trustworthiness of the findings. All transcripts were included in the analysis, with data saturation achieved. One staff participant checked the findings to enhance the credibility of the findings.

### Results

Fifteen adolescents participated in three FGs (mean age = 13.7 years; standard deviation = 1.9; 53% female; 60% from Black and minority ethnic groups; 33% living in the top three deciles for deprivation), consisting of 4–6 participants and an average duration of 60 min (range: 56–64 min). Interviews were conducted with eight staff members, consisting of six school staff and two catering staff (75% female; 37.5% aged 35–44 years), with an average duration of 42 min (range: 29–55 min). Table 1 displays participant characteristics.

**Table 1:** Adolescent and staff participant characteristics

Adolescent participants					
ID	Age	IMD decile^a^	Gender	Ethnic group aggregated	Focus group
A1	13	1	Female	Not White British	Focus Group 1
A2	13	6	Male	White British	Focus Group 1
A3	13	2	Female	Not White British	Focus Group 1
A4	13	4	Female	Not White British	Focus Group 1
A5	12	7	Female	White British	Focus Group 1
A6	16	7	Female	Not White British	Focus Group 2
A7	17	7	Male	White British	Focus Group 2
A8	18	4	Male	Not White British	Focus Group 2
A9	14	2	Female	Not White British	Focus Group 2
A10	14	7	Male	White British	Focus Group 2
A11	14	8	Male	White British	Focus Group 2
A12	12	4	Male	Not White British	Focus group 3
A13	12	5	Male	White British	Focus group 3
A14	12	2	Female	Not White British	Focus group 3
A15	12	1	Female	Not White British	Focus group 3

Staff participants					
ID	Age		Gender	Role	
C1	35–44	n/a	Female	Catering staff	n/a
C2	35–44	n/a	Female	Catering staff	n/a
S1	25–34	n/a	Female	School staff	n/a
S2	45–54	n/a	Male	School staff	n/a
S3	25–34	n/a	Female	School staff	n/a
S4	45–54	n/a	Female	School staff	n/a
S5	55–64	n/a	Male	School staff	n/a
S6	45–54	n/a	Female	School staff	n/a

^a^IMD decile of home postcode. 1 = most deprived decile

A summary of the results of thematic analysis related to study objectives 1–3 is provided, with example quotes provided in Table 2. Table 3 presents a summary of findings relating to objective 4. The coding tree is provided in Additional file 3.

**Table 2:** Themes and example quotes relating to themes

Theme	Adolescent quotes	Staff quotes
Autonomy and informed decisions	*“And it’s got to be all about choice, you’ve got to choose. It’s, but then a bit of subconscious influences around, like, putting the healthy stuff closer to you but you’ve got to choose what you want.”* A10 *“No because if there’s no, like, if there’s no unhealthy stuff wouldn’t people just bring unhealthy stuff from, from, like, home?”* A12 *“You could, like, almost kind of, like, lie and say, like, that they’re sweet potato chips and then, like, people, like, but it’s actually carrots. And then people end up actually liking it and then they get more.”* A13	*“I think that there are, you know, there are lots of misconceptions about what makes something healthy and what makes something nutritious. And I think sometimes kids have, you know, bad ideas, they’ve been told things that are incorrect about nutrition.”* C2 *“They’d see straight through it [nudge] and wouldn’t buy it.”* C1
Value	*“You could just buy the cakes rather than wasting money on, like, a salad.”* A11	*“Well kids love them [fruit] for 10p. They’re not gonna pay 25p for it but they’ll pay 10p. And I’m like… if I give fruit away with every meal then maybe they would eat healthier in that respect.”* C1
Presentation	*“I think if, like the healthy food looked nicer then people would, like, give it a try.”* A9 *“If you’re gonna buy something you should know what’s inside and you should have a clear view on it.”* A7	n/a
Adolescents’ taste preferences and valuing of predictability	*“When they taste nice it helps because you just kind of like you want to eat it, you’re not eating it ‘cause you have to”* A14 *“You know, like, if you bring something from home it’s something you like. 'Cause you don’t know necessarily what you’re gonna get in the canteen. But you know what you’re gonna bring in from home.”* A10	*“[Pupils] aren’t particularly interested in the nutritional value of what they’re eating, it’s more about is it food that they want to enjoy?…”* S5 *“It [a healthy lunch] means that they, that the children eat, because it doesn’t matter how healthy and nutritious I make something, if they don’t eat it it’s not nutritious because they’re not eating it.”* C2
Lunchtime is about more than just food	*“I think, people want to get into the canteen, have their, like, get their food and then go out so they can have an actual lunchtime. And if they walk in and pasta and the fruit is next to them, or whatever, the healthy food is next to them, I think they’ll be more inclined to go for that”.* A10	*“One, they don’t want to sit down. It depends on what their friends are doing as well. They don’t want to miss out on their social time. They don’t want to miss out in case they go up, some of their friends go up to the fields and they start a game of football and they’re going to be late and they’re going to miss out on half of the game. I think, I think time is a, is a lot to do with them not eating a healthy meal.”* S4
Canteen-based barriers to a healthy school lunch	*“One, it’s like really busy and there’s like loads of people just getting their food. Like it’s kind of cramped sometimes.”* A8 *“…now our canteen is, like, people don’t, aren’t really responsible. And say if, like, there’ll probably be theft. And then setting up café style and, like, add toppings to your salad, that’ll probably just get really messy and people will be really careless.”* A1	*“Right now, those dining halls are so crammed and so small, that’s why the kids won’t eat in there, that’s why they want to grab and go and go to the next thing. They don’t have the time to sit and eat and they don’t have an environment that they want to sit and eat in..”* C2 *“it is an ongoing concern that students who come to school and will, will take food, and it’s not just because I think they’re, they’re, you know, they’re inherently wanting to thieve, I think it’s just that they, they either lack the self-regulation and/or they’re hungry.”* S5 *“The problem is that you try to do it, you take them away [‘treat’ foods], then the kids don’t buy anything and so therefore they, we lose money and it’s not viable and, you know*” C2
Competing influences	*“Because then you’ve got people surrounding you as well with, like, again, food from the canteen. They will have, like, sometimes they’ll have less healthier snacks than you, and then it’ll make you feel like, “Okay, maybe I should have this ‘cause everyone else has it.” And it’ll kind of, like, make you, like, not want to be as healthy.”* A4	*“The new guidelines aren’t like that, so they’re interpretative, so, you know, I might interpret them slightly differently than someone else, and they might interpret them slightly different to someone else. And then we all get told off by the same person who’s interpreted them slightly differently. And at the moment there’s no regulation and there’s no one to give the definitive interpretation of those.”* C2

**Table 3:** Summary of adolescent and staff participant views relating to each intervention strategy, organised by intervention category

Category	Strategy	Adolescent views (appeal)	Staff views (feasibility)
Availability	Wider choice of salads/vegetable dishes	Adolescents value choice	Report high demand for wide choice Some cynicism about pupils purchasing salads Concern about increased food waste
	Ban on sales of unhealthy snacks and drinks within school canteen	Removes choice/autonomy (viewed negatively) Belief that teens need sugary foods for energy boost Pupils would purchase banned foods elsewhere	Easily implemented in canteen However, pupils would purchase banned foods elsewhere
Position	Unhealthy snacks placed behind till, available upon request only	Viewed as likely to be effective because ‘Out of sight, is out of mind’	Viewed as likely to be effective since pupils prioritise speed Concerns about lack of space behind tills Potential consequence is increased desirability of hidden food items
	Fridge reorganisation (healthier drinks more prominent)	Viewed as likely to be effective because ‘Out of sight, is out of mind’	Already in place to some extent so easy to implement
Functionality	Colour coded serving utensils (to indicate whether to have large or small amounts of each dish depending on calorie content)	Viewed as helpful and instructive However, would slow service down	Low cost to implement However, would slow service down
	Express/self-service till for healthy food items only	Viewed as likely to be effective as adolescents value speed Concerns about behavioural issues (i.e. theft)	High initial cost outlay for infrastructural changes Expect high levels of pupil buy-in as suits their desire for speed Concerns about behavioural issues (i.e. theft) Concerns about high volume of customers removing the ‘express’ nature of the strategy Requires a large amount of space
	Pre-ordering of lunchtime meal	Viewed negatively as removes opportunity for spontaneity However, adolescents believed it allows for more considered/rational choices	Resource-intensive for catering staff to implement High start-up costs for pre-order electronic system Volume of customers too high to be practical in secondary school setting
	‘Cash for cookies’ (treat foods cannot be purchased using pre-paid cards^a^)	Viewed as fundamentally unfair to specific groups of pupils e.g. children from low income families Adolescents expect this would encourage unhealthy purchases as cash purchases are unmonitored by parents (as opposed to cashless systems) Adolescents had concerns about carrying cash (i.e. theft)	Administrative cost in re-introducing cash-based system too high Would lead to a loss of valuable data about purchases via the cashless system
Presentation	Pre-chopped fruits and vegetables	Viewed as visually appealing and on-trend	Viewed as an economical use of left-over produce
	Salad toppings station	Viewed positively as adolescents value choice Concerns about behavioural issues (i.e. mess)	Expect high levels of pupil buy-in Some cynicism about pupils purchasing salads Concerns about a high volume of pupils using it
	Dining room decoration to improve ambience	Would create a more visually appealing environment for eating	Requires only an initial cost outlay, so relatively inexpensive to implement
	Café style set-up (food service) to improve presentation/appeal of food purchasing environment	Appealing aesthetic, creates an inviting purchasing environment Concerns over behavioural issues if foods were presented so openly (i.e. theft)	Viewed as appealing to young people Viewed positively as mimics out-of-school environment Concerns over behavioural issues if foods were presented so openly (i.e. theft)
	Guided floor markings e.g. footprints to healthy food/drink items	Viewed as fun However, dining room too crowded so wouldn’t be visible	Dining room too crowded so wouldn’t be visible Viewed as more appropriate for primary school children
Size	Smaller plates (to make portion sizes appear larger)	Concerned about hunger due to smaller portion sizes Viewed as too manipulative	Low cost so inexpensive to implement Concerned about student resistance as pupils would not want smaller portion sizes
Information	Simple traffic light label scheme	Adolescents value the opportunity for more informed decision-making However, adolescents prioritise taste	Scheme would need to be supported by education/curriculum learning Concerns about maintaining an accurate database of nutritional information for all dishes
	Promotional posters to encourage healthy eating	Adolescents value the opportunity for more informed decision-making Adolescents expect reduced impact/visibility over time	Low cost so inexpensive to implement
	Pupil taste tests of new healthy dishes	‘Try before you buy’ approach viewed as reducing risk of wasting money on disliked dishes Provides greater autonomy to adolescents to make informed choice based on taste preference	Viewed as appealing to pupils Easy to organise
	Nutritional information available on mobile app (e.g. nutritional content of dishes at point of purchase; and/or post-purchase individualised report of nutritional intake)	Viewed as increasing autonomy through self-monitoring of purchases However, adolescents critical of the potential additional screen time required Adolescents reported potential for negative peer-peer competitive consumption as an unintended consequence	Would work well within existing cashless payment system (has this functionality) Expect high levels of parent buy-in Mixed views on expected levels of pupil buy-in
	Social media promotion of healthy dishes	Disliked the potential for dishonest visual representations of dishes	Reluctance to open up food provision to pupils’ feedback over online platform (potential for abuse) Concerns about increasing pupils’ social media use
	Verbal prompt from lunch staff to add fruits/vegetables	Adolescents felt this added unnecessary pressure to select certain items, which may not be eaten/wasted	Considered easy to implement as can be built into normal conversations with pupils with no additional resources required

^a^Pre-paid cards/systems are commonly used in UK school canteens operating a ‘cashless system/school’. Parents/guardians pre-load cards with money for in-school purchases (for those receiving Free School Meals, their entitlement is also pre-loaded)

#### Theme 1. Autonomy and informed decisions

Staff felt a nudge approach would be appropriate in a secondary school setting because adolescents had little knowledge of nutrition/healthy food choices, so needed to be supported to make the right choices. There was a conflict in adolescents, between a desire to make informed decisions for themselves, and acknowledgement that they sometimes need to be “tricked” into making healthy choices. Both adults and adolescents referred to the idea of being “tricked” ambivalently. The line appeared to be drawn differently depending on the child’s age, with a belief (from staff) in the need for increasing autonomy in decision-making for older adolescents.

#### Theme 2. Value for money

Adolescents and staff agreed that pricing was usually an important factor in children’s lunch choices. Young people want to feel full after lunch, and will opt to get more food (quantity) for the same price when possible. Adolescents felt healthy food was more expensive, which discouraged healthy selections.

#### Theme 3. Food and drink presentation

Presentation was viewed as influential upon food choices for adolescents. Food needs to look appealing and ingredients need to be visible in dishes/on packaging to avoid any unwanted surprises in their meal.

#### Theme 4. Adolescents’ taste preferences and valuing of predictability

For adolescents, taste was prioritised. ‘Unhealthy food’ e.g. pizza (in adolescents’ descriptions) was viewed as more flavoursome, and there was high demand for these types of foods. Adolescents and staff agreed that healthy food would be more appealing if it tasted better. Caterers felt that in order to create appealing meals for adolescents, the healthiness of dishes had to be compromised to some extent.

Adolescents were viewed by adults as being reluctant to try new foods, which was echoed by adolescents reporting that they felt it was a high-risk option to try something new. Staff also felt that school was a setting in which adolescents could broaden their tastes but this contrasted with pupils’ expectations that a canteen should provide familiar, preferred foods.

#### Theme 5. Lunchtime is about more than just food

Adolescents viewed lunchtime primarily as a time to spend with friends, with eating as a secondary activity. This view appeared to drive adolescents’ beliefs and behaviours around purchasing habits, e.g. the desire for speed and convenience (the so-called “grab and go culture”); and the negative views of the canteen as a space to be in.

#### Theme 6. Canteen-based barriers to a healthy school lunch

Lunch service was considered too short in duration to enable healthy choices, and adolescents felt their choices were often rushed and poorly thought-out. The canteen was viewed as an unappealing space—hectic and crowded, with too many teachers present (observing; disciplining), and too little space for all pupils to have a sit-down meal. Another barrier was the competing demands upon caterers to balance the provision of healthy food with other factors e.g. minimising waste; profitability. Although the canteen was seen as part of the school ‘community’, with a moral purpose to provide healthy lunches to pupils, staff acknowledged that it was primarily a business, and needed to be viable. Healthy food items were viewed by some staff as less profitable, mainly because of low take-up and high levels of waste.

#### Theme 7. Competing influences

Staff felt that other, broader factors had a larger influence on adolescents’ diets than the school setting e.g. home; society.

It was felt that one consequence of providing fewer ‘unhealthy’ options at school (e.g. cakes, cookies, pizza) was that customer numbers would decline as pupils sought these items from off-site outlets. For staff, the canteen was considered preferable to off-site outlets, since there was some degree of control over the nutritional content and purchasing of less healthy items on the school site.

The School Food Standards (SFS) were influential in restricting the sale of ‘non-compliant’ items e.g. sugary drinks. However, the SFS were viewed as open to interpretation, making implementation a challenge. The SFS did not appear as influential in the sixth form setting, since non-compliant items were available to buy in sixth-form-only spaces. This was considered appropriate since older teens are more able to make responsible choices and need to be exposed to such food environments to prepare them for the outside world.

Caterers viewed themselves as the driving force behind making healthy choices available to schools. There appeared to be no external incentive for schools to provide a healthy lunch to adolescents, other than the school’s own values/approach, which caterers found to vary widely across schools.

#### Attitudes towards specific nudge strategies and healthy eating messages

Views regarding specific nudge strategies are summarised in Table 3, categorised by type. The most feasible and appealing strategies were within the “position”, “presentation” and “information” domains, with students additionally finding “availability” strategies appealing. The potential efficacy of some nudge strategies relying on presentation, information and positioning appeared to be reduced by the volume of pupils using the canteen in this school, reducing the visibility of, and obstructing access to, food counters and information. Many nudge strategies were considered unsuitable by both adolescents and staff because they provided additional opportunities for behavioural problems e.g. theft; mess.

The most appealing healthy eating messages were those that were short, factual and memorable. Messages focused on physical appearance or those that evoked feelings of guilt (e.g. “eat something good without feeling bad”) were unpopular, viewed as unfair or stigmatising by adolescents. Messages that were positive or focused on feeling good (e.g. “choose well, feel great”) were more appealing. Adolescents appeared to be influenced negatively by social pressure, and a reluctance to stand out/deviate from the norm, which discouraged healthy eating. For adolescents and staff, the motivation to eat healthily was that a healthy meal provides fuel for learning. This tended to be focused on the need for volume, to ‘fill’ pupils up, but also extended to nutrient density and a balance of food groups.

### Critical discussion

This study adds to our knowledge of the perceived drivers of adolescent food choices in the school canteen: convenience, presentation and value for money. In addition the findings highlight the perceived barriers to implementing nudge strategies imposed by the school canteen environment, e.g. short lunchbreaks; large volumes of customers; the need to achieve financial viability.

The current study identified position strategies (to make the healthy options the most convenient) as having high acceptability and feasibility. This supports other qualitative research in this age group [5]. Our study suggested that increased choice and availability of healthy items was highly acceptable to adolescents, supporting previous findings that the most *effective* interventions in increasing vegetable purchases/consumption were those where the variety was increased [6]. However, our findings provide some insight into the practicality of implementing such strategies. Caterers in the current study suggested that this approach would be a challenge to implement, due to the risk of increased waste and impact on financial viability. Two types of messaging appeared motivating for adolescents: messages that highlight how healthy choices support learning; and marketing strategies that focus on getting a large quantity of food for a low price.

The findings of this research have two potential uses in the design of future interventions: (1) identifying strategies that appear practically feasible to implement; and (2) building a theoretical underpinning for understanding why some strategies may be more effective than others in this population and setting, which will support the evaluation of any future intervention.

### Conclusions

The study suggests that the general idea of ‘nudging’ for dietary change in a school canteen is acceptable to secondary school pupils, school staff and caterers, but that any choice architecture intervention implemented in a secondary school needs to be tailored to this age group and the setting to maximise successful implementation.

## Limitations

These findings come from a limited number of participants all recruited from one school, so may not generalise to other schools. FGs incorporated pupils across mixed age groups, which may have impacted on the findings e.g. 13–15 year olds were generally less active in discussion when older pupils were present; and 11–13 year olds were generally the most enthused by the strategies proposed. Despite achieving a diverse sample, there may be some sampling bias due to pupils being selected by a teacher. We were only able to test a limited number of specific strategies, and have attempted to say something about intervention types more generally. Additional testing of a wider range of specific strategies within each of the most promising ‘categories’ of intervention types is needed. On this basis, the current study is a starting point for qualitatively exploring acceptability and feasibility.

## Supplementary Information

**Additional file 1.** Semi-structured topic guides for focus groups and interviews

**Additional file 2.** Summary of messages tested in focus groups with adolescents

**Additional file 3.** Coding tree for thematic analysis

## Data Availability

The datasets generated and/or analysed during the current study are not publicly available as we do not have explicit participant consent to publish transcripts (which may contain identifiable data), but are available from the corresponding author in abridged and anonymised form on reasonable request.

## Abbreviations

FG: Focus group
IMD: Index of multiple deprivation
SFS: School Food Standards
