# Supplementary material for: Acceptability and feasibility of strategies to promote healthy dietary choices in UK secondary school canteens: a qualitative study
Source: BMC Res Notes. 2021 Sep 20;14:365. doi: 10.1186/s13104-021-05778-3 (PMC8454098; doi:10.1186/s13104-021-05778-3)
Supplement: Supplementary file 1 — Additional file 1. Semi-structured topic guides for focus groups and interviews [file 13104_2021_5778_MOESM1_ESM.docx]

Additional file 1. Semi-structured topic guides for focus groups and interviews

| Questions for adolescents |
| --- |
| Ice breaker  What makes a healthy school lunch?  How much does this resemble your school lunch choice on a day-to-day basis?  What makes it easy to eat a healthy school lunch?  What gets in the way of eating a healthy school lunch?  Verbal description of nudge  How do you feel about this approach?  Card sorting activity 1: nudge strategies  Tell me about the ones you liked. Why do you like these?  Tell me about the ones you disliked. Why do you dislike like these?  Card sorting activity 2: messages  Tell me about the ones that appeal to you. Why do they appeal?  Tell me about the ones that didn’t appeal to you. Why were they unappealing? |
| Questions for staff |
| What does a ‘healthy school lunch’ mean to you?  How important is school food?  How easy is it for pupils to have a healthy school lunch in your school/schools?  What does your service/school do to support children to have a healthy lunch?  What are the important factors when designing your menu? How are the choices made?  Verbal description of nudge  How do you feel about this approach to changing food behaviours?  Card sorting activity 1: nudge strategies  Why do you think these things are easy to implement?  Why do you think these things are difficult to implement?  How would you measure the impact of these strategies upon pupil’s food selections? |
