# Supplementary material for: Acceptability and feasibility of strategies to promote healthy dietary choices in UK secondary school canteens: a qualitative study
Source: BMC Res Notes. 2021 Sep 20;14:365. doi: 10.1186/s13104-021-05778-3 (PMC8454098; doi:10.1186/s13104-021-05778-3)
Supplement: Supplementary file 2 — Additional file 2. Summary of messages tested in focus groups with adolescents [file 13104_2021_5778_MOESM2_ESM.docx]

Additional file 2. Summary of messages tested in focus groups with adolescents

1. Calorie-equivalent labelling (number of minutes physical activity required to expend calories)
2. Eat something good without feeling bad poster
3. Train hard Eat Fresh message delivered by famous sportsperson (poster)
4. Good for You label
5. Fruit is fast food poster
6. Sandwiches with a little bit extra: get more in your sandwich
7. Did you know? Eating an apple is a more reliable method of staying awake than consuming a cup of coffee
8. Let’s get sugar smart (Change4Life campaign poster)
9. You just ate 16 packs of sugar (picture of a soft drink). All those extra calories can bring n obesity, diabetes and heart disease.
10. Choose well. Feel great. Traffic light labelling poster (Best choice = Green; choose carefully = amber; Limit = red)
11. Photograph of a group of teens eating watermelon
12. Picture of healthy dishes with Facebook ‘likes’
13. Comic strip promoting healthy messages
14. Reach a new height. Students who eat a daily breakfast score higher on tests and are better at problem solving. Eat smart. Be smart.
15. Don’t ask why healthy food is so expensive. Ask why junk food is so cheap.
16. Healthy eating head-to-toe. Fuel your body. Alongside image of teen linking foods to improved health for brain, hair, eyes, heart, digestive system, skin, muscle and bones.
17. Image of athlete kicking an oversized burger, milkshake, doughnut and chocolate
18. Eat right and the pants won’t feel tight message
