# Supplementary material for: Acceptability and feasibility of strategies to promote healthy dietary choices in UK secondary school canteens: a qualitative study
Source: BMC Res Notes. 2021 Sep 20;14:365. doi: 10.1186/s13104-021-05778-3 (PMC8454098; doi:10.1186/s13104-021-05778-3)
Supplement: Supplementary file 3 — Additional file 3. Coding tree for thematic analysis [file 13104_2021_5778_MOESM3_ESM.docx]

Additional file 3. Coding tree for thematic analysis

Major interpretative themes for thematic analysis (objective 1 – 3)

Codes pertaining to attitudes related to specific nudge strategies or specific messaging (objective 4)
